# Levofloxacin-Induced Acute Pancreatitis

**DOI:** 10.7759/cureus.13301

**Published:** 2021-02-12

**Authors:** Tiago Neto Gonçalves, Débora Sousa, Natália Marto, Alexandra Bayão Horta

**Affiliations:** 1 Internal Medicine Department, Hospital da Luz Lisboa, Lisbon, PRT

**Keywords:** pancreatitis, antibiotic, levofloxacin, quinolone, adverse reaction, side effect

## Abstract

Drug-induced pancreatitis is a rare entity accounting for less than 2% of acute pancreatitis (AP). Quinolones are commonly used antimicrobials with occasional reports of pancreatitis. We present the case of a 74-year-old man who was diagnosed with acute cystitis five days before hospital admission and was treated with levofloxacin (LVF). Two days after initiating LVF he experienced fever, severe abdominal pain, and nausea. The initial assessment revealed leukocytosis, elevated C-reactive protein, and a significant elevation of amylase and lipase. On abdominal ultrasound, the head of the pancreas revealed an hypoechogenic region suggestive of inflammatory edema. A diagnosis of AP was established. The drug was withdrawn along with supportive care, with complete resolution of the symptoms. No other probable causes of AP were found after further investigation. Although rare, LVF-induced pancreatitis should be considered when managing a patient with AP. Increasing physician awareness is vital to the prompt recognition of this entity.

## Introduction

Drug-induced acute pancreatitis (DIAP) is a rare entity accounting for less than 2% of acute pancreatitis (AP) cases [1]. Most cases follow a mild or moderate course, usually with a good prognosis. However, severe and even fatal cases can occur. Failure to recognize the offending drug can result in important delays and adverse outcomes [2]. The diagnosis is challenging for clinicians and requires ruling out more common etiologies. Drugs likely to cause AP should be discontinued or exchanged for an alternative medication. The resolution of disease with these measures further increases the suspicion of DIAP. More than 500 drugs that can cause AP are listed in the World Health Organization’s database. Quinolones are commonly used antimicrobials that inhibit bacterial DNA-gyrase. The association between ciprofloxacin, norfloxacin, and gatifloxacin and AP is well established, and classified by Badalov DIAP classification system as class Ib, IV, and IV, respectively [3]. To the best of our knowledge, there are only three case reports of pancreatitis induced by levofloxacin (LVF) [4-6]. Here, we report the case of a patient who developed AP while being treated with LVF.

## Case presentation

A 74-year-old man with a known history of benign prostatic hyperplasia reported dysuria, urinary frequency, and suprapubic pain five days before admission to our hospital ward. A diagnosis of acute cystitis was established and LVF was started. The urinary symptoms partially resolved, but two days after initiating LVF, he experienced fever (39ºC), severe epigastric pain radiating to the back, nausea, and malaise. The patient denied any other medication or over-the-counter substance use, alcohol consumption, recent copious meals, or abdominal trauma. There was no personal history of biliary lithiasis or any identical episode in the past and the family history was negative for pancreatic disease. No recent travels were identified. On physical examination, he was hemodynamically stable and afebrile, with hypoactive bowel sounds and epigastric tenderness on palpation.

Initial assessment revealed a white blood cell count of 10.20 × 10^9^/L, with 86% neutrophils (8.77 × 10^9^/L) and a C-reactive protein level of 83.3 mg/L. A four-fold elevation of amylase (419 U/L) and 10-fold elevation of lipase (4,705 U/L) were also found. Liver enzymes and renal function were within normal range.

An abdominal ultrasound showed a non-distended gallbladder without edema or wall thickening. The head of the pancreas revealed an hypoechogenic region suggestive of inflammatory edema without signs of biliary tract obstruction. A diagnosis of AP was established.

To investigate common causes of AP, an abdominal magnetic resonance (MR) and an MR cholangiopancreatography were undertaken. No biliary sludge or lithiasis were found, excluding the hypothesis of calculous pancreatitis. There were no anatomical abnormalities such as annular pancreas or pancreaticobiliary malunion, nor any evidence of malignancy. Other etiologies such as hypertriglyceridemia (patient had a normal level of 1.1 mmol/L) and hypercalcemia (patient had low ionized calcium of 1.11 mmol/L) were also excluded. Autoimmune causes were not investigated due to the patient’s advanced age and lack of other suggestive manifestations. Infectious etiologies were also evaluated and excluded. Blood cultures, hepatotropic virus (hepatitis C and B virus) and human immunodeficiency virus serologies were negative. Due to the absence of clinical symptoms suggestive of a viral infection, other viruses were not investigated.

After admission, hydration with isotonic crystalloid solution, analgesics, antiemetics, and *nil per os* were initiated. Considering lack of other probable causes, a relationship between pancreatitis and the recent introduction of LVF was considered. No other drug was recently initiated. LVF was withdrawn and replaced by trimethoprim-sulfamethoxazole on the second day of hospitalization.

The patient had a complete resolution of the symptoms allowing to start a low-fat diet. No organ dysfunction was perceived. Laboratory tests improved, as shown in Table 1.

**Table 1:** Laboratory test results. WBC, white blood cell; CRP, C-reactive protein

	Admission	Day one	Day two	Day four
Hematocrit (%)	41.5	37.8	37.7	38.4
WBC (×10^9^/L)	10.20	8.16	10.77	7.68
CRP (mg/L)	83.3	133.2	224.0	92.0
Lipase (U/L)	4,705	2,727	-	-

The patient was discharged five days after admission. During follow-up at three and six months, the patient remained asymptomatic with no recurrence.

## Discussion

In 2001, Mennecier et al. [4] first described a case of DIAP in a 31-year-old woman 48 hours after initiating LVF and methylprednisolone for acute sinusitis. In this case, the development of pancreatitis only two days after beginning treatment suggests LVF to be the most probable cause. In 2008, Jiménez et al. [5] presented a case of a 57-year-old woman with AP two days after initiating antibiotic therapy with LVF for infected bronchiectasis. Recently, Moreno et al. [6] reported another case of AP induced by LVF in a 28-year-old female with Kartagener syndrome who started LVF for a respiratory infection. Almost 48 hours after beginning treatment with LVF, she presented with acute abdominal pain and a diagnosis of DIAP was established. In all these reports, other etiologies were accurately searched and excluded.

The precise mechanism behind LVF-induced AP is not well-known. Most of the proposed mechanisms for DIAP pathophysiology have not been proved yet. LVF-induced AP may probably result from an idiosyncratic reaction such as pancreatic duct constriction, cytotoxic and metabolic effects, accumulation of a toxic metabolite or intermediary, and hypersensitivity reactions [3,7].

Our case report shows a consistent latency [1] of symptom onset with previous reports. Other probable causes based on age and clinical manifestations were excluded. We also verified a positive dechallenge. No rechallenge procedure was done due to ethical reasons. Based on causality assessment of adverse drug reactions by Edwards et al. [8], we classify this event as probable/likely.

## Conclusions

This case highlights LVF as a potential cause of AP. Although rare, in a patient who has recently started this drug and presents with AP, prompt recognition of this etiology is warranted, and the drug should be withdrawn.
